# On Beri-Beri in Asylums

**Published:** 1899-10-14

**Authors:** 


### ON BERI-BERI IN ASYLUMS.

Dr. Conolly Norman,[1] who has had considerable experience of the above disease, which has recently been prevalent in the Richmond District Asylum at Dublin, gives an interesting account of the epidemic, with a record of cases, and of clinical facts observed.

Until a few years ago the appearance in Europe of beri-beri, which had not been immediately and directly imported, was a thing unheard of. Many people have not even yet realised what the researches of physicians in Northern Japan and Saghalien have clearly demonstrated, that beri-beri is not necessarily a tropical disease. Its prevalence in Brazil shows that it is not exclusively an Eastern disease, while its recrudescence in that country after a long period of quiescence seems to indicate that it may be in that condition of increased activity which the history of other epidemics shows may occur from time to time without any as yet understood reason.

Within the last five years epidemics or sporadic outbreaks of beri-beri have appeared in asylums for the insane in Ireland, England, France, and North America. Of these epidemics the first broke out in the Richmond District Asylum at Dublin in summer 1894; another in the Suffolk County Asylum at Melton in winter 1894. Epidemics have also occurred at the Alabama and Arkansas State Asylums, U.S.A., in 1895; and an outbreak was recorded at the St. Gemmes-sur-Loire Asylum in France in the summer of 1897. The early cases at Dublin in 1894 were insidious in onset, and not very severe in character. "A few isolated cases occurred in the early summer; then there was a gradual increase, then a very great and sudden increase (September, 1894), then almost a quick falling off, and no fresh cases occurred after October." In the first year 127 men and 47 women, or a total of 174 inmates, were affected. The registered deaths from this cause were 18 men and 7 women; total, 25. The disease was remarkably more prevalent among the epileptics than among other patients. It is to be observed that in its relation to season, and in its incidence among the sexes, this epidemic followed the ordinary habits of beri-beri. No cases were observed in 1895. It reappeared in July, 1896, and 114 persons (31 male and 76 female patients and 7 nurses) were affected; 2 male and 6 female patients died). None of the nurses succumbed. In 1897 246 cases occurred; of these 45 were male patients, 193 female patients, 2 male attendants, and 6 nurses; 3 male and 8 female patients died. During the year 1898 12 cases occurred among women, 4 of whom succumbed to the affection. Some of these cases were first attacks, some had suffered before. In most cases death occurred from slow decay, and, apparently, was due to fatty heart, consecutive to the more acute forms of disease. Cases that had survived previous attacks seemed to suffer from feeble and irritable heart, and, if allowed to get up, soon showed signs of œdema, feebleness of gait, and sensory troubles. At the Tuscaloosa Aslyum, Alabama, the disease began with the following symptoms. Some cases had fever and gastro-intestinal irritation at the outset. In other instances the attack was insidious, it being impossible to date the commencement of the attack. In others, again, the initial symptom was suddenly occurring dyspnœa with tachycardia and violent pulsation of the vessels of the neck; while in others œdema of the feet and ankles was the first indication. In Alabama, as in Dublin, the epileptics suffered most severely in the first epidemic. The same thing was observed in the Saint-Gemmes Asylum in France. The first thing in the latter institution to attract attention was an unusual mortality among the epileptics, who died apparently in the *status epilepticus*, and whose bodies were found to present a considerable degree of œdema. Attention having been drawn to this condition, it was found that a number of other patients —epileptics, melancholics, and idiots—suffered from dropsy without presenting the usual causes of that symptom. The œdema had the peculiarity of being more solid than is usual in the ordinary forms of dropsy, cardiac and renal; the same peculiarity was observed in the patients affected at the Suffolk County Asylum. The œdema usually began about the feet and ankles, and subsequently spread to such an extent as to involve the whole body, including the serous cavities. Paralysis of the lower extremities with foot-drop soon followed; the inner edge of the foot being slightly raised, and the dorsum arched in the position characteristic of peripheral neuritis. Paralysis sometimes extended to the superior extremities, and also to the diaphragm, death in the latter event supervening from asphyxia. The paralyses were accompanied by extreme muscular atrophy, and by remarkable sensory disturbances, tenderness of muscles, cramps, formication, and cutaneous anæsthesia, or by hyperæsthesia, and the patellar reflexes were abolished. Tachycardia was a prominent symptom, the pulse ranging from 140 to 150, and becoming weakened and almost imperceptible at the wrist. There was generally no fever, except such as was secondary to bedsores, &c. Vomiting and bronchorrhœa were common symptoms. There was no anorexia, but the reverse. The essential cause of the disease is as yet obscure.

[1] Journal of Mental Science, July, 1899.

